# COVID-19 vaccine coverage targets to inform reopening plans in a low incidence setting

**DOI:** 10.1098/rspb.2023.1437

**Published:** 2023-08-30

**Authors:** Eamon Conway, Camelia R. Walker, Christopher Baker, Michael J. Lydeamore, Gerard E. Ryan, Trish Campbell, Joel C. Miller, Nic Rebuli, Max Yeung, Greg Kabashima, Nicholas Geard, James Wood, James M. McCaw, Jodie McVernon, Nick Golding, David J. Price, Freya M. Shearer

**Affiliations:** ^1^ Population Health and Immunity Division, WEHI, Parkville 3052, Vic, Australia; ^2^ School of Mathematics and Statistics, The University of Melbourne, Melbourne, Victoria, Australia; ^3^ Melbourne Centre for Data Science, The University of Melbourne, Melbourne, Victoria, Australia; ^4^ Centre of Excellence for Biosecurity Risk Analysis, The University of Melbourne, Melbourne, Victoria, Australia; ^5^ Centre for Epidemiology and Biostatistics, Melbourne School of Population and Global Health, The University of Melbourne, Melbourne, Victoria, Australia; ^6^ Department of Infectious Diseases, The University of Melbourne, Melbourne, Victoria, Australia; ^7^ School of Computing and Information Systems, The University of Melbourne, Melbourne, Victoria, Australia; ^8^ Victorian Infectious Diseases Reference Laboratory Epidemiology Unit at the Peter Doherty Institute for Infection and Immunity, The University of Melbourne, Melbourne, Victoria, Australia; ^9^ Department of Econometrics and Business Statistics, Monash University, Clayton, Victoria, Australia; ^10^ Infectious Disease Ecology and Modelling, Telethon Kids Institute, Perth, Western Australia, Australia; ^11^ Department of Mathematics and Statistics, La Trobe University, Melbourne, Victoria, Australia; ^12^ School of Population Health, The University of New South Wales, Sydney, New South Wales, Australia; ^13^ Quantium, Sydney, New South Wales, Australia; ^14^ Curtin School of Population Health, Curtin University, Perth, Western Australia, Australia

**Keywords:** COVID-19, SARS-CoV-2, vaccination strategy, mathematical modelling, public health policy, pandemic response

## Abstract

Since the emergence of SARS-CoV-2 in 2019 through to mid-2021, much of the Australian population lived in a COVID-19-free environment. This followed the broadly successful implementation of a strong suppression strategy, including international border closures. With the availability of COVID-19 vaccines in early 2021, the national government sought to transition from a state of minimal incidence and strong suppression activities to one of high vaccine coverage and reduced restrictions but with still-manageable transmission. This transition is articulated in the national ‘re-opening’ plan released in July 2021. Here, we report on the dynamic modelling study that directly informed policies within the national re-opening plan including the identification of priority age groups for vaccination, target vaccine coverage thresholds and the anticipated requirements for continued public health measures—assuming circulation of the Delta SARS-CoV-2 variant. Our findings demonstrated that adult vaccine coverage needed to be at least 60% to minimize public health and clinical impacts following the establishment of community transmission. They also supported the need for continued application of test–trace–isolate–quarantine and social measures during the vaccine roll-out phase and beyond.

## 1. Introduction

In early 2020, Australia adopted a strong suppression strategy in response to the COVID-19 pandemic, aiming for no community transmission of the SARS-CoV-2 virus [1]. As a result of a broadly successful implementation of this strategy, which included international and internal travel restrictions, much of the population lived in a COVID-19-free environment up until late 2021. Nonetheless, sporadic outbreaks and a number of major (but geographically isolated) waves of infection occurred, most notably the ‘second wave’ in the state of Victoria in June–October 2020 [2] and the Delta ‘third wave’ seeded into New South Wales in June 2021 [3], which quickly spread into neighbouring jurisdictions.

Like elsewhere, the availability of highly effective vaccines for COVID-19 from early 2021 provided a new opportunity to protect the population and reduce harms related to SARS-CoV-2 [4]. However, the transition from a state of minimal (daily and cumulative) incidence and low vaccine coverage to one of high vaccine coverage and established but manageable transmission presented unique challenges compared to vaccination roll-out in high incidence and high pre-existing immunity settings.

In much of Europe and North America, where COVID-19 has circulated widely since its emergence and both daily and cumulative incidence were high at the time of vaccine availability, vaccination provided a very clear, although challenging, ‘re-opening’ pathway. As vaccine campaigns were initiated, with ancestral and Alpha variants in circulation, increasing vaccine coverage reduced the ability of the virus to spread and assisted in bringing case incidence down [5]. While the emergence of the Delta variant led to a resurgence in cases, there was clear evidence that vaccination played an important role in ‘decoupling’ clinical caseloads and deaths from mild infections [6].

By contrast, for countries such as Australia, where pre-vaccination cumulative incidence was very low and the population remained largely susceptible, the relationship between vaccination, incidence and the public perception of COVID-19 was markedly different. Any plan to ‘re-open’ society and transition away from a strong suppression strategy would necessarily lead to an *increase* in cases, morbidity and mortality, with clear challenges for communication and decision making. The ‘National Plan to transition Australia's National COVID-19 Response’ (hereafter the National Plan) [7], released in July 2021, describes a transition from ‘phase A’ in which strong suppression and no community transmission is the goal, to ‘phase B’ whereby SARS-CoV-2 infection was allowed to establish in the broader population. This transition was enabled by vaccination. The aim was for COVID-19 to be manageable, from both a public health and clinical perspective, through continued but more targeted application of public health and social measures (PHSMs) and test–trace–isolate–quarantine (TTIQ) strategies. The targets in the National Plan were agreed by all Australian States and Territories at National Cabinet (forum for the prime minister, state premiers and chief ministers to meet) on 6 August 2021. The transition from phase A to phase B was the first step on the path to ‘re-opening’. Latter phases ‘C’ and ‘D’ in the National Plan were supported by higher vaccine coverage levels allowing for further reductions in whole-of-population pandemic responses. Exploring target coverage thresholds for vaccination to enable the transition from phase A to phase B, and the required level of supporting interventions including ongoing PHSMs and TTIQ capabilities was identified as a priority under the National Plan. More broadly, identifying such thresholds was a common challenge for low incidence settings other than Australia [8,9].

Here, we report on the dynamic modelling study used to identify priority age groups for vaccination, target coverage thresholds, and the anticipated requirements for continued PHSMs and TTIQ to support a transition to a more open society in which SARS-CoV-2 could circulate while the health and clinical impacts remain manageable. Findings from this research directly informed specific re-opening policies within the National Plan and in particular the threshold and conditions for the transition from phase A to phase B.

## 2. Modelling framework

**Figure 1.**
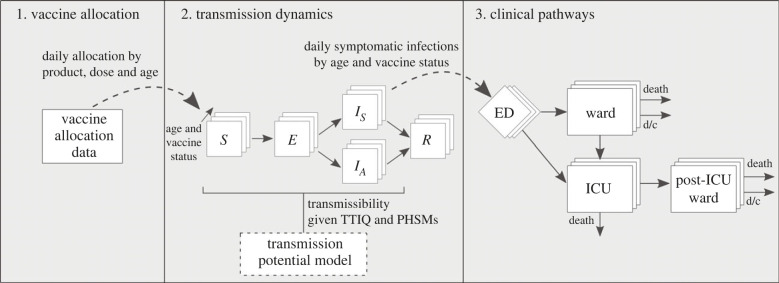
Schematic of three interlinked model components. 1. Data on vaccine allocation over time to the Australian population (aged 16 and over). 2. An age-structured IBM of SARS-CoV-2 transmission dynamics to model infection numbers by age and vaccine status. Note that the transmissibility of SARS-CoV-2 under different PHSM bundles and TTIQ capabilities is informed by outputs from a model of SARS-CoV-2 transmission potential described in [10]. 3. A clinical-pathways stochastic model to map infection numbers to hospital admissions, ward and ICU occupancy, and deaths, stratified by age and vaccine status. *S*, susceptible; *E*, exposed; *I_S_*, infectious and develops symptoms; *I_A_*, infectious and does not develop symptoms; *R*, recovered; ED, emergency department; ICU, intensive care unit; d/c = discharge.

The modelling framework comprises three distinct components (depicted in figure 1), each summarized here and described in detail in the electronic supplementary material.

— A model of vaccine allocation and roll-out to the Australian population (aged 16 and over).
— An age-structured individual-based model (IBM) of SARS-CoV-2 transmission dynamics to model infection numbers by age and vaccine status.
— A clinical-pathways stochastic model to map infection numbers to hospital admissions, ward and intensive care unit (ICU) occupancy, and deaths.

These three components are underpinned by an analysis of SARS-CoV-2 transmissibility in Australia, measured by the ‘transmission potential’ (TP). As described elsewhere [11], the TP draws on a number of behavioural data streams to estimate (at a state level) the reproduction number that could be expected during widespread transmission. The TP is distinct from the effective reproduction number in that it represents the expected reproduction number of a pathogen in the general population rather than the reproduction number among active cases (who may not be representative of the general population). The TP is included as an indicator in Australia's national COVID-19 surveillance plan [12] and thus routinely reported for all states and territories of Australia and made publicly available through the Australian Government's Common Operating Picture [13]. Under pre-pandemic conditions, the TP corresponds to the basic reproduction number, *R*_0_. It varies through time due to changes in health system performance, different public health orders, and trends in population mixing and behaviours (such as limiting non-household contacts and adherence to cough etiquette and other infection control recommendations). Furthermore, because vaccines act to reduce transmission (through a reduction in the probability of acquisition and reduced contagiousness given breakthrough infection), additional reductions in TP due to vaccination can be estimated in the context of behavioural and public health response settings.

Using the TP model and time-series data on cases and population behaviours in Australia since March 2020, we first conducted a static analysis (described in [14]), to estimate TP for the Delta variant achieved under alternate vaccine allocation strategies, PHSMs and TTIQ capabilities. An example output from this analysis is displayed in figure S1 of the electronic material (adapted from Ryan *et al.* [14]). For the static analysis, we considered four ‘bundles’ of PHSMs: baseline, low, medium and high. Each bundle relates to a specific time and place in Australia's pandemic experience, thereby capturing both behavioural responses and the proportional reduction in TP achievable by PHSMs in the Australian context (table 1 for details).

**Table 1.** Description of measures implemented under different ‘bundles’ of public health and social measures (PHSMs). Each bundle relates to a specific time and place in Australia's pandemic experience up to mid-2021—thereby capturing behavioural responses and the proportional reduction in ‘transmission potential’ (TP) achievable by PHSMs in the Australian context. The proportional reductions in TP observed at each time and place can therefore be related to similar reductions achieved via other combinations of PHSMs (not limited to the bundles in place during the reference period). Similarly, the imposition of any given combination of PHSMs at different times and places may result in variable population responses and thus reductions in TP. NSW, state of New South Wales; VIC, state of Victoria.

PHSM bundle	description
baseline	minimal density/capacity restrictions and no major outbreaks, as in NSW March 2021
low	more stringent capacity restrictions compared to baseline (e.g. hospitality venues limited to 10 customers per booking), as in NSW 23 August 2020
medium	stringent capacity restrictions, group size limits (e.g. fewer than five people outdoors), stay-at-home orders (except work, study, essential purposes), as in NSW 1 July 2021
high	no household visitors, curfew, stay-at-home orders (except essential purposes and permitted work), schools closed (remote learning only), as in VIC 23 August 2020

The static analysis also considered the contribution of TTIQ in reducing virus spread, which is described in detail in [15]. Briefly, this included a detailed study of a limited time-series of case data from New South Wales between July 2020 and January 2021. During this time, caseloads in New South Wales were low and the impact of TTIQ was clear in suppressing transmission [11]. The empirical distribution of times from case detection to isolation was determined and used to evaluate the reduction in transmission due to TTIQ. This reduction—54%—defines an ‘optimal’ TTIQ effect, representing the maximum reduction in TP that we may expect due to TTIQ at any time during an epidemic. By assuming improvements in TTIQ are proportional to improvements in times to detection (i.e. times from symptom onset to test), the relative performance of TTIQ was measured during different periods of epidemic activity. This approach provided a distribution of times to isolation in epidemiological contexts where TTIQ was assessed to be partially effective. In particular, when calibrated against the data for the state of Victoria from 4 August 2020—the peak of daily locally acquired COVID-19 cases in Australia in 2020—this gives an estimated reduction of 43% in TP and defines the ‘partial’ TTIQ scenarios explored in the static analysis.

For all scenarios considered in this study, the dynamic transmission model uses a baseline reproduction number of 6.32, corresponding to a TP estimated for the Delta variant under baseline PHSM and after ‘backing out’ the effect of TTIQ (indicated by the dashed line ‘Baseline TP’ in figure S1 in the electronic supplementary material). The baseline TP is lower than the *R*_0_ for SARS-CoV-2 due to the baseline behavioural changes in the Australian population. It is further reduced based on the assumed impact of (partial or optimal) TTIQ, enhanced (low, medium or high) PHSMs and vaccination.

Here, we examine epidemic dynamics and clinical consequences of infections following transition from phase A to phase B of the National Plan (table 2) at different vaccine coverage thresholds between 50% and 80%, for alternative age-based vaccine allocation strategies, and assuming continued application of PHSMs and TTIQ.

**Table 2.** Phases of the ‘National Plan to transition Australia's National COVID-19 Response’ [7]. Our modelling analysis focuses on the transition from ‘phase A’ in which strong suppression and no community transmission is the goal, to ‘phase B’ where vaccine coverage is high and SARS-CoV-2 infection is allowed to establish in the population. Scenarios therefore examine the epidemic dynamics and clinical consequences of infections following seeding of an epidemic at different vaccination prioritization strategies and coverage thresholds.

phase	description	activities
A	vaccinate, prepare and pilot	continue to strongly suppress the virus for the purpose of minimizing community transmission
B	vaccination transition	seek to minimize serious illness, hospitalization and fatality as a result of COVID-19
C	vaccination consolidation	as above
D	post-vaccination	manage COVID-19 consistent with public health management of other infectious diseases

### (a) Vaccine allocation strategy and timing of the roll-out

Vaccine prioritization in the Australian population through the first half of 2021 was based on a direct-protection approach, targeted towards those most at risk of severe outcomes. Two products were approved for distribution: AstraZeneca (ChAdOx1 nCoV-19) and Pfizer/BioNTech (BNT162b2 (mRNA)). By 11 July, based on Australian Immunisation Register (AIR) data, 33% of the population had received one-dose and 11% two-doses of a licensed vaccine (details provided in electronic supplementary material, table S2). This low starting point provided substantial scope to explore the importance of age cohort coverage within overall targets of 50–80% uptake in the population aged 16 years and over.

From this baseline position, four vaccine allocation strategies, and associated delivery scenarios, were considered: ‘Oldest first’, ‘40+ years first’, ‘All adults’, and an implementable strategy consistent with the national COVID-19 immunization programme designated the ‘Transmission reducing’ strategy. Table 3 provides details for these alternative allocation strategies. The population-level impacts of these strategies are strongly related to underlying assumptions for age-specific mixing, susceptibility and infectiousness. A detailed description of these assumptions and their interaction with differential age cohort coverage under the target thresholds is provided in the electronic supplementary material and [14].

**Table 3.** Vaccine allocation strategies by age group, assuming July 2021 recommendations in Australia for AstraZeneca vaccine age eligibility (60+ years) and dosing interval (12 weeks). Within each age group, vaccines are allocated at random according to the Australian Government's in-house agent-based vaccine allocation model.

strategy	allocation sequence
oldest first	vaccines are prioritized from oldest to youngest
	specifically, prioritization occurs in the following order: 80+, 70–79, 60–69, 50–59, 40–49, 30–39, 20–29, 16–19
40+ years first	vaccines are prioritized from 40+ upwards, then 16+
	specifically, prioritization occurs in the following order: 40–49, 50–59, 60–69, 70–79, 80+, 16–19, 20–29, 30–39
all adults	vaccines are not prioritized in any particular order by age
transmission reducing	as for national programme, under which all individuals aged 40+ are eligible at 8 June 2021; within the simulation time frame, the 30–39-year-olds cohort becomes eligible from 30 August 2021, and 16–29-year-olds on 11 October 2021

Within the constraints of available supply (at the time of analysis, July/August 2021), AstraZeneca is provided to those aged 60+ years at a dosing interval of 12 weeks. Pfizer/BioNTech is provided to those aged 16–60 years at a dosing interval of three weeks. For both vaccines, a two-week delay from second dose completion to full efficacy was assumed.

The daily allocation of vaccines by product, dose, and age group, for each strategy were provided by the Australian Government Department of Health and directly fed into the transmission dynamics model. These vaccine allocation datasets were a combination of actual vaccine coverage data and modelled outputs. The modelled outputs were generated in-house by the Australian Government Department of Health. Briefly, they employed an agent-based model using location and allocation data on vaccination sites and location data for the Australian population. Each week a subset of the population seeks vaccination (according to the allocation strategy's age-based eligibility criteria) at available sites within their respective geographical area. Sites receive deliveries of vaccines and administer vaccinations to the seeking population up to their level of stock. Age prioritization occurs in the order of the respective prioritization strategy. For example, under the ‘oldest first’ strategy, each region will prioritize the vaccination of the 80+ age group first before moving on to the 70–79 age group, then the 60–69 age group, and so on. Within each age group, vaccines are allocated at random according to the Australian Government's in-house agent-based vaccine allocation model. Figure S1 of the electronic supplementary material presents the modelled two-dose vaccine coverage time-series by age group, which explicitly visualizes how vaccines were rolled out to age groups under each of the four allocation strategies, with the modelled terminal vaccine coverage by age group and strategy displayed in table S3 of the electronic supplementary material. In all scenarios considered below, the vaccine allocation model outputs full time-series by vaccine type (Pfizer, AstraZeneca) and dose (dose 1 and dose 2), which are fed into the transmission dynamics model.

For any given coverage threshold considered for the transition from phase A to phase B in the National Plan, the TP is computed (methods detailed in the electronic supplementary material and [14]). This differs by allocation strategy as vaccine product (Pfizer, AstraZeneca) varies by age, and the timing between doses varies for Pfizer and AstraZeneca. Table S6 of the electronic supplementary material presents the achieved TP at the key eligible-population (16+ years) coverage thresholds of 50%, 60%, 70% and 80%. These values define the initial transmissibility of SARS-CoV-2 in subsequent simulations of epidemic activity as now described. Vaccination continues to roll-out during these simulations according to the mean modelled output from the allocation model.

### (b) Transmission model

We developed an age-structured IBM of SARS-CoV-2 transmission dynamics, calibrated to the Australian population. Panel 2 of figure 1 presents a simplified state-diagram of the epidemiological-status for an individual in the synthetic population (see electronic supplementary material for a more detailed schematic). Individuals in the population may be susceptible to infection, partially protected due to vaccination (with multiple sub-classes depending on vaccine type, number of doses received and time since last vaccination), exposed, infectious or recovered. Among those infectious, the model distinguishes between those displaying symptoms or otherwise. We further assume that recovered individuals are 100% protected against reinfection and neither infection- nor vaccine-induced immunity wanes over time. While evidence available at the time of analysis showed that reinfections were rare but possible—including from a large healthcare worker cohort study in England which estimated the median interval between SARS-CoV-2 infection and reinfection to be more than 200 days (in the pre-Delta era) [16]—we did not expect waning of immunity to significantly impact our results over the timescale explored, particularly given Australia's highly limited exposure history.

Age-specific mixing, susceptibility and transmissibility assumptions employed in the dynamic transmission model are the same as those used in the static analysis [14]. Briefly, population mixing within and between age groups is configured based on synthetic social contact matrices published by Prem *et al.* [17], expanded to include an 80+ age class (assumed to have the same mixing rates as 75–79 years). Estimates of age-specific susceptibility and symptomatic fractions from Davies *et al.* [18] are used to compute an age-specific transmission matrix calibrated to the population-wide TP (electronic supplementary material, figure S4). The highest transmission rate is anticipated between individuals aged from 15 to 24 years, remaining high through adults of working age. While intense school-based mixing is anticipated between children aged 5–14, the transmission matrix embeds a relatively low infectiousness of this age group, due to the high proportion of asymptomatic infections (as estimated by Davies *et al.* [18]) and an assumed 50% relative infectiousness of asymptomatic individuals. These linked assumptions and parameter estimates are taken from Davies *et al.* [18] who calibrated age-specific infectiousness and susceptibility parameters against infection age distributions from non-immune populations in six countries in early 2020. The age-specific contributions to TP accounting for demography, relative susceptibility and transmissibility, and vaccine coverage for overall coverage levels (16+) of 50%, 60%, 70% and 80% under the four allocation strategies (oldest first, 40+ years first, all adults, and transmission reducing) are examined in [14].

COVID-19 vaccines have been shown to act on multiple elements of transmission and disease. Vaccine parameters related to onward transmission in breakthrough infection and symptomatic infection—for the Delta variant [6,19]—are detailed in tables S4 and S13 of the electronic supplementary material, respectively. We assume that vaccination reduces susceptibility to infection (according to electronic supplementary material, table S4, left column) and the probability of developing symptomatic disease given infection (according to electronic supplementary material, table S13). The latter impacts transmission since we assume that asymptomatic individuals are 50% less infectious. We further assume that infected vaccinated individuals are less infectious by a factor calculated to match combined vaccine effectiveness assumptions on transmission (electronic supplementary material, table S4, right column).

We use a baseline reproduction number of 6.32, corresponding to a TP estimated for the Delta variant under ‘baseline’ PHSMs and after ‘backing out’ the effect of TTIQ (indicated by the dashed line ‘Baseline TP’ in figure S1 of the electronic supplementary material). The static analysis of vaccine impacts on TP [14] indicated that even 80% coverage under an optimal allocation strategy was unlikely to achieve a control TP of 1. We therefore consider the overlaid impacts of differing degrees of PHSMs and TTIQ in the dynamic transmission model. The application of low and medium PHSMs are considered in the IBM through modification to the baseline reproduction number as estimated by Ryan *et al.* [14]. Partial and optimal TTIQ are incorporated by sampling the isolation time for each infected individual from the corresponding distribution of times from infection to isolation as estimated by Shearer *et al.* [15].

We seed simulations with a fixed low number of infections at the time the vaccine coverage threshold is reached, reflecting a scenario in which the virus re-establishes itself in the Australian population via a border incursion or is allowed to enter given the achieved vaccine threshold. We seed with a sufficient number of infections (30 unvaccinated people by default) to exclude (with high probability) the chance of stochastic extinction. Despite Australia's strong suppression strategy, in late June 2021 the Delta variant had established itself in the community in New South Wales and Victoria. By late August 2021 (the time of these analyses) daily case incidence was in the thousands in New South Wales, in the hundreds in Victoria, and low or zero in all other jurisdictions. Accordingly, we also examined scenarios in which the initial number of infections is ‘high’ (thousands of infections), ‘medium’ (hundreds of infections) or ‘low’ (tens of infections). While each of these initial conditions is low by global standards, they were highly pertinent to the Australian context and re-opening plan. For these medium and high initial conditions, we seed epidemics with a mix of vaccinated and unvaccinated individuals. Cumulative incidence remains negligible in both situations at the time of reaching the coverage threshold and so scenarios remain equivalent in terms of the susceptible population size. To aid comparisons in this additional analysis of initial seeding size, we also re-run the ‘low’ scenario, simulating tens of infections in both vaccinated and unvaccinated individuals. Further technical details of the IBM construction and initialization approach are provided in the electronic supplementary material.

### (c) Clinical pathways

Similar to previous studies [20,21], we model hospital admissions, ward/ICU occupancy and death. The model takes inputs of daily symptomatic infections, stratified by age and vaccine status, from the transmission dynamics model. A fraction of those with symptomatic infection, either vaccinated or unvaccinated, will present to hospital and require admission for additional care. Patients admitted to hospital may occupy either a ward or ICU bed. Ward stays may also deteriorate and require ICU care, before returning to a general ward and discharge. Death as an endpoint may also occur while admitted, during either a ward or ICU stay. These flows are represented using a stochastic model, with age-specific transitions and length-of-stay distributions informed by international data [20]. Details are provided in the electronic supplementary material. Transitions through the clinical-pathways model for both unvaccinated and vaccinated individuals are calibrated to the Alpha variant due to a lack of available data on the Delta variant at the time of our analysis. Clinical severity parameters for the Alpha variant are displayed in tables S9 and S10 of the electronic supplementary material [22–26]. Vaccine parameters related to clinical outcomes (hospitalization, ICU admission, death) for the Alpha variant are displayed in table S13 of the electronic supplementary material [4,6,27–32]. We note that around the time of initial reporting of our findings to policymakers, evidence of Delta's increased severity (in both vaccinated and unvaccinated individuals) was beginning to emerge, and we reflect on the consequences of this in the Discussion. Our analysis does not account for health system capacity constraints, and so outputs represent the anticipated demand for clinical services. Thus, if a scenario was to exceed health system capacity, our simulations would underestimate clinical burden since individuals who are unable to access care will likely have worse outcomes. Further, we do not indicate whether health system capacity would be exceeded under a given scenario, since capacity is difficult to pre-define, highly dynamic and potentially misleading to represent at a national level. However, please refer to our jurisdictional-level modelling analysis which addresses the complexity of this issue in the context of jurisdictional population size and related health service resourcing [33].

### (d) Sensitivity analysis

We conduct a *post hoc* sensitivity analysis by exploring the impact of more optimistic and pessimistic parameters for clinical severity and vaccine effectiveness, given epidemic seeding at 50%, 60%, 70% and 80% vaccine coverage. All simulations are under the transmission reducing vaccination strategy, seeded with a fixed, low number of infections (30 unvaccinated people), and assuming baseline PHSMs and partial TTIQ. For the sensitivity analysis of vaccine effectiveness parameters, we explore the impact of increasing (optimistic) and decreasing (pessimistic) all vaccine effectiveness values by 10%.

As previously stated, the primary analysis was conducted and reported to decision-makers in mid-2021 when data on clinical severity of the Delta variant were lacking, hence our ‘Baseline’ severity parameters are calibrated to the Alpha variant. For the sensitivity analysis of clinical severity parameters, we therefore use estimates of clinical severity for the Delta variant as of October 2021 (i.e. from studies published *after* reporting of results from the primary analysis). We refer to this parameter set as ‘updated delta’ severity. We also explore the impact of more optimistic clinical parameters by assuming that severity was equivalent to ancestral SARS-CoV-2 virus (‘Ancestral’ severity). Values for each parameter set are provided in tables S4, S9 and S11–S13 of the electronic supplementary material.

### (e) Graphical presentation

Figures displaying time-series of epidemiological quantities computed from the transmission and clinical-pathways models show the 90% confidence interval of trajectories calculated across time (i.e. the 5- and 95-percentiles on each day) as coloured ribbons. A single representative trajectory is also shown to give an indication of the dynamics within each scenario. The single trajectory within each scenario was selected as corresponding to the median total infections (or symptomatic infections, ward occupancy, ICU occupancy, deaths, as appropriate) across the projection horizon.

## 3. Results

**Figure 2.**
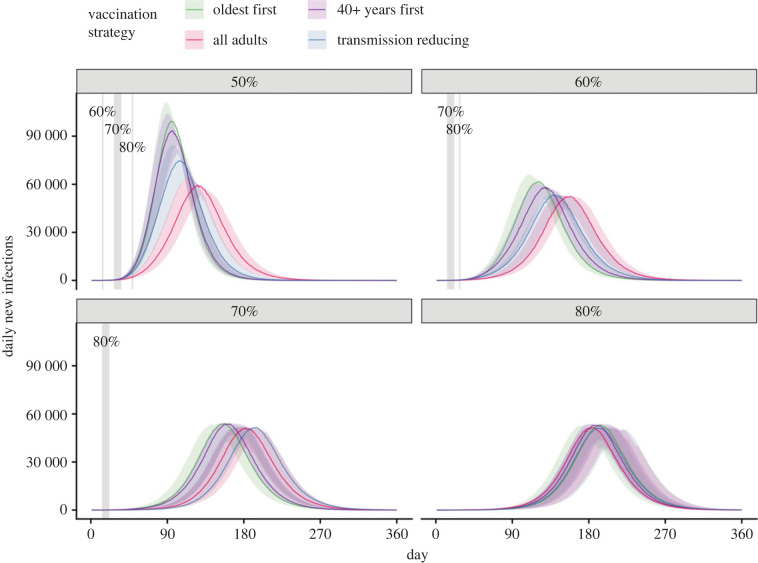
Comparison of time-series for infections (asymptomatic and symptomatic) by vaccine allocation strategy and vaccine coverage threshold for transition from the National Plan phase A to phase B. All simulations were seeded with a fixed, low number of infections (30 unvaccinated people). Solid coloured lines and coloured shading represent median epidemic trajectories and 95% confidence intervals, respectively. Vaccination continues to roll-out beyond the target threshold during each simulation according to the mean modelled output from the allocation model. Solid grey vertical lines (or shading) indicate the date of achieving each vaccine coverage threshold.

A comparison of time-series for daily infections, by vaccine allocation strategy and vaccine coverage threshold for transition from phase A to phase B, is presented in figure 2. The corresponding time-series for ward and ICU occupancy, and deaths, are displayed in figures S7–S10 of the electronic supplementary material, and cumulative infections and deaths are displayed in figures S11 and S12 of the electronic supplementary material. Here, baseline PHSMs and partial TTIQ are assumed to be in place. These analyses demonstrate that the ‘All adults’ and ‘Transmission reducing’ strategies result in fewer infections (figures 2 and electronic supplementary material, figure S11) and afford greater clinical protection (electronic supplementary material, figures S8–S10 and S12) compared to the ‘Oldest first’ and ‘40+ years first’ strategies at all transition coverage levels. Furthermore, marked reductions in the time to epidemic peak and peak size are noted as vaccine coverage increases from 50% to 60% and beyond.

The ‘Transmission reducing’ strategy was designed as an implementable version of the ‘All adults’ strategy and is considered hereafter. Furthermore, when our initial results (shown in figure 2 and electronic supplementary material, figures S7–S10) were considered alongside a complementary analysis that specifically considered the time in lockdown and economic consequences for different vaccine thresholds (see [14] for a description of the modelling analysis and [23] for the government report), they indicated that adult vaccine coverage of least 70% was required to support a transition compatible with Australia's strategic road map [10]. We therefore now restrict our focus to vaccine coverage thresholds of 70% and above.

**Figure 3.**
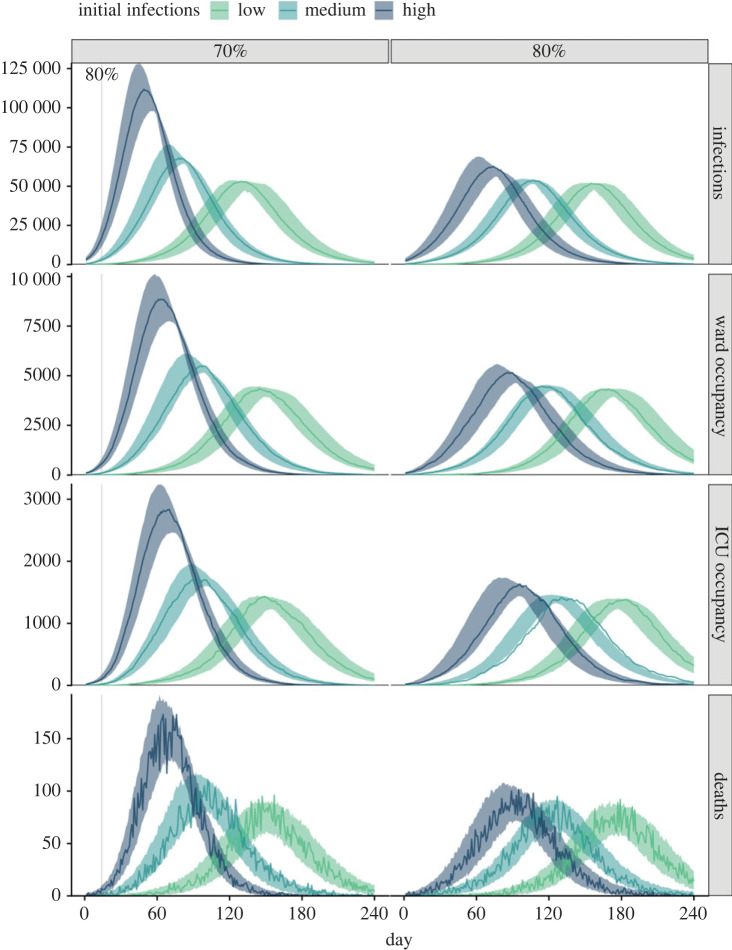
Time-series of infections (asymptomatic and symptomatic), occupied ward and ICU beds, and deaths for epidemics seeded with low (tens), medium (hundreds) and high (thousands) numbers of initial infections at the 70% (left) and 80% (right) coverage thresholds. All scenarios assume baseline PHSMs and partial TTIQ are applied. Solid coloured lines and coloured shading represent median epidemic trajectories and 95% confidence intervals, respectively. Vaccination continues to roll-out beyond the target threshold during each simulation according to the mean modelled output from the allocation model. Solid grey vertical lines indicate the date of achieving the 80% vaccine coverage threshold. ICU, intensive care unit; PHSMs, public health and social measures; TTIQ, test–trace–isolate–quarantine.

In recognition of the Delta variant becoming established in some Australian jurisdictions prior to reaching 70% coverage thresholds, we examine the consequences of an increased case load at the time of transition from phase A to phase B in the National Plan. Row one of figure 3 presents time-series of infections (asymptomatic and symptomatic combined) for three initial conditions following transition from phase A to phase B at either 70% (left) or 80% (right) coverage. Baseline PHSMs and partial TTIQ are applied. For a transition at 70% coverage, an increase from ‘low’ (tens) to ‘medium’ (hundreds) numbers of infections at the time of transition results in a marked leftward shift in the timing of the epidemic. This result is unsurprising given basic epidemic theory. However, if the transition occurs at a point with ‘high’ (thousands) infections, the epidemic is not only left-shifted, but peak and final size also increase. This is a result of dynamic ‘overshoot’, otherwise avoidable due to the continued roll-out of vaccines and hence increasing coverage level during the epidemic. If the transition from phase A to phase B is made at 80% coverage, the leftward shift in epidemic dynamics remains, but even for the ‘high’ (thousands) infections scenario, there is minimal impact on peak and overall size of the epidemic. This is due to two factors: (i) vaccine coverage is sufficiently high to prevent a large ‘overshoot’; and (ii) under the model for vaccine distribution, the rate of increase in coverage slows beyond 80% as we approach saturation of vaccine uptake in the eligible population (around 89.9% of the eligible population, reached at 56 weeks). Corresponding time-series for clinical outcomes are presented in rows two to four of figure 3.

**Figure 4.**
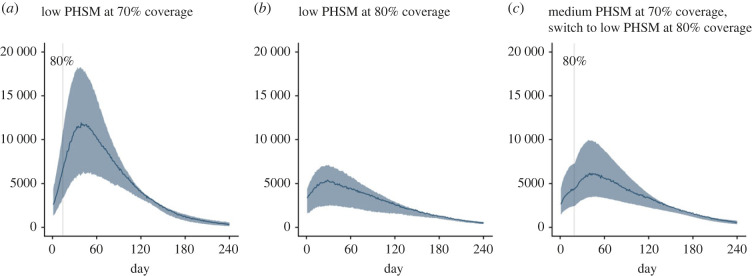
Time-series of infections (asymptomatic and symptomatic) when low PHSMs are applied from the point of the transition from phase A to phase B of the National Plan at 70% and 80% vaccine coverage (*a*,*b*). (*c*) Demonstrates an adaptive strategy where medium PHSMs are applied during the period from 70% to 80% coverage, easing to low PHSMs thereafter. All scenarios assume ‘high’ numbers of initial infections and the application of partial TTIQ. Solid coloured lines and coloured shading represent median epidemic trajectories and 95% confidence intervals, respectively. Vaccination continues to roll-out beyond the target threshold during each simulation according to the mean modelled output from the allocation model. Solid grey vertical lines indicate the date of achieving the 80% vaccine coverage threshold. PHSMs, public health and social measures; TTIQ, test–trace–isolate–quarantine.

Australia's National Plan envisages continued application of enhanced PHSMs to further suppress epidemic activity and minimize morbidity and mortality. Figure 4*a*,*b* demonstrates the marked benefit of continued application of low PHSMs, accompanied by partial TTIQ, from the point of transition from phase A to phase B. At both 70% (figure 4*a*) and 80% (figure 4*b*) the epidemic is strongly suppressed compared to under baseline PHSMs (figure 3). Similar beneficial outcomes can be achieved through application of optimal TTIQ under baseline PHSMs (electronic supplementary material, figure S13), although we note that maintaining optimal TTIQ was not considered feasible over the long term.

While all of the above simulations assume a fixed policy from the point of transition from phase A to phase B, substantial benefits may be realized by the imposition of increased restrictions for limited periods of time. Figure 4*c* shows how an adaptive strategy can support a transition at 70% even under ‘high’ numbers of infections. Medium PHSMs are applied during the period from 70% to 80% coverage, with low PHSMs enacted thereafter. Compared to the scenario in which low PHSMs are active during this transition period (figure 4*a*), infections are notably reduced. Overall epidemic impact is similar to when the transition from phase A to phase B is only made at 80% (figure 4*b*).

**Figure 5.**
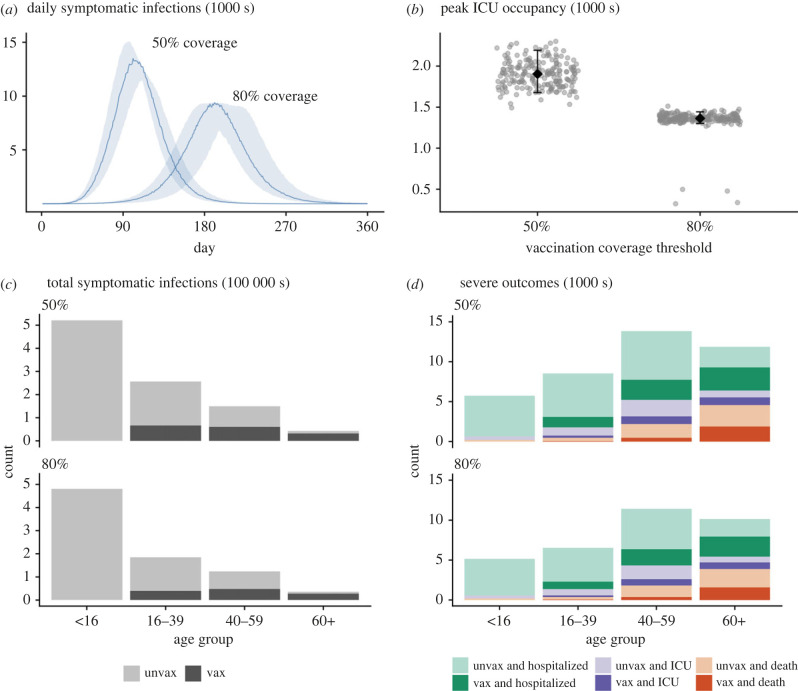
Comparison of symptomatic infections and clinical impacts given transition from phase A to phase B leading to established community transmission at coverage thresholds of 50% and 80% for the ‘Transmission reducing’ strategy, assuming baseline PHSMs and partial TTIQ. All simulations were seeded with a fixed, low number of infections (30 unvaccinated people). Vaccination continues to roll-out beyond the target threshold during each simulation according to the mean modelled output from the allocation model. (*a*) Time-series of symptomatic infections (solid lines = median epidemic trajectory; shading = 95% confidence intervals). (*b*) Peak ICU occupancy (grey dots = estimate for each model simulation, black diamonds and bars = median and 5th and 95th percentiles). (*c*) Number of cumulative symptomatic infections by age group and vaccine status. (*d*) Number of cumulative ward admissions, ICU admissions and deaths by age group and vaccine status. Unvax, unvaccinated; Vax, vaccinated; ICU, intensive care unit; PHSMs, public health and social measures; TTIQ, test–trace–isolate–quarantine.

Figure 5 compares the cumulative number of symptomatic infections and clinical impacts by age group and vaccine status for outbreaks seeded at 50% and 80%, assuming baseline PHSMs and partial TTIQ. There is a significant overall reduced burden given establishment of community transmission at 80% compared to 50% coverage (figure 5*a*,*b*). In this context, a substantial fraction of symptomatic infections and severe outcomes are anticipated in vaccinated individuals within highly vaccinated age groups. At both 50% and 80% vaccine coverage, a decoupling of infections from clinical burden is evident, with infections concentrated in younger less vaccinated populations, yet much greater health impacts are anticipated in older more highly vaccinated age groups (figure 5*c*,*d*).

Results of our *post hoc* sensitivity analysis exploring the impact of more optimistic and pessimistic parameters for clinical severity and vaccine effectiveness, given epidemic seeding at 50%, 60%, 70% and 80% vaccine coverage thresholds, are shown in figures S14–S20 of the electronic supplementary material. The patterns in epidemic dynamics between coverage thresholds for alternate parameters sets are not markedly different from those seen in the primary analysis.

## 4. Discussion

To transition from a strategic goal of ‘no community transmission’ to one of ‘minimizing COVID-19 burden’ requires sufficient vaccine coverage to (i) suppress case incidence such that TTIQ remains an effective response to reduce transmission and (ii) ensure anticipated health and clinical impacts remain manageable. Here, through a model-based analysis, we have demonstrated the requirement for adult vaccine coverage of at least 60% using the ‘Transmission reducing’ allocation strategy to achieve these objectives. This result assumes the continued application of TTIQ (partial) and PHSMs (baseline) during the vaccine roll-out phase and beyond. These dynamic results were considered by policy makers alongside a complementary analysis that specifically considered the risks of escalating case numbers under different vaccine thresholds, requiring re-imposition of lockdown with associated economic consequences (see [14] for the modelling analysis and [34] for the government report). Based on the combined analysis of health and economic risks, a threshold adult vaccine coverage of least 70% was determined as the target to support a transition compatible with Australia's strategic road map [10]. Our analyses were conducted based on assumed circulation of the Delta variant of SARS-CoV-2 and contingent upon the Australian epidemiological, health system and societal context in which circulation of SARS-CoV-2 was strongly suppressed up to the point of transition.

If, as was the case in the state of Victoria, case incidence was high (thousands per day) at the time of reaching the 70% coverage threshold, then heightened but temporary public health and social measures (medium PHSMs) could help bridge the period to achieving 80% coverage, reducing the risk of a surge in transmission that may threaten capacity. With coverage at 80% or beyond, our analyses indicate that epidemic dynamics would likely be manageable within the constraints of the clinical system and only baseline PHSMs and partial TTIQ in place, compatible with Australia's strategic roadmap for managing COVID-19 in the vaccine era. Further gains—in terms of reduced infections and so reduced hospitalizations and deaths—can be afforded by maintenance of low PHSMs over the vaccine roll-out phase and beyond. Furthermore, those low PHSMs would support a strong and more effective TTIQ response, helping avoid escalation of local epidemic activity. If the transition to ‘living with COVID’ were to occur prior to reaching 70% coverage, case numbers would likely rise to such a level that TTIQ effectiveness was diminished and epidemic ‘overshoot’ would result in additional—and *a priori* avoidable—cases, hospitalizations and deaths.

Our findings are comparable to those from model-based studies for other low prevalence settings [8,9]. Nguyen *et al.* [8] and Steyn *et al.* [9] investigated the impacts of alternative age-based vaccine allocation strategies and coverage thresholds in the New Zealand context. Similar to Australia, New Zealand's vaccine roll-out was intended to support a shift in response strategy from elimination to border re-opening and virus circulation. Both studies concluded that high vaccine uptake (e.g. greater than 80% of the population aged 16+) and maintenance of other public health measures during the vaccine roll-out phase would be required to prevent serious adverse health impacts. Furthermore, studies from high prevalence settings, including the United Kingdom, also highlighted the potential adverse health and health system impacts of complete relaxation of social restrictions during the early phases of vaccine roll-out [4]. Of course, our specific findings on vaccine coverage thresholds are limited by the low level of transmission in Australia prior to vaccine roll-out and are not generalizable to higher prevalence settings.

A key limitation of our work is that we considered a single, large population (24 million) in which the virus spreads. This was a deliberate and necessary choice designed to support the Australian national (whole-of-country) re-opening plan. However, and particularly in the early establishment phase of a country wide epidemic, transmission was expected to be highly focal. Jurisdictions where SARS-CoV-2 transmission established prior to the 70% coverage threshold, such as New South Wales and Victoria, began a transition from a state of ‘medium’ and ‘high’ case incidence, respectively (electronic supplementary material, figures S21 and S22). Other jurisdictions maintained zero case incidence well beyond vaccine coverage thresholds of 70% and 80%. Furthermore, at a sub-jurisdictional level, we would expect systematic differences in vaccine coverage, behavioural patterns and TTIQ capabilities. These considerations emphasized the need for small-area assessment of TP (to anticipate risk) and other real-time epidemiological metrics.

More broadly, the goal of scenario analyses such as those reported here is to provide insights on potential patterns in epidemic activity given different assumptions about the future, including what intervention options are chosen. Scenario analyses are not forecasts or predictions of the future course of the epidemic, not least because of uncertainty in key model inputs, including vaccine effectiveness, the duration of immunity, intrinsic severity of the Delta variant and future population behaviour. When we reported the findings documented in this manuscript to government in mid-2021 to support Australia's COVID response strategy, ongoing situational assessment (as described elsewhere [11,35]) was acknowledged as critical to the success of the National Plan. That is, monitoring of local data was anticipated to allow benchmarking of the scenarios to guide real-time policy decision making on the transition to phase B of the National Plan. Likewise, a summary of the scenario modelling on vaccination and the easing of restrictions in the United Kingdom, published in February 2021, articulated the need for measures to be relaxed ‘based on data and the situation at the time, rather than at pre-determined dates' [4]. The degree of PHSMs needed for disease control prior to the vaccine threshold being reached in Australia and during the transition period would require reference to near-real-time estimates of the effective reproduction number, and forecasts of cases and clinical burden, at a sub-national level. Outcomes were anticipated to be highly situation specific—related to the actual starting number of cases, the population characteristics where transmission is concentrated (e.g. vaccine coverage, age, co-morbidities, access to health services, ability to adhere to personal protective measures, etc.), the rate of vaccination, and the level of epidemic suppression achieved.

In mid-2021 transmission of the Delta variant became established prior to the 70% coverage threshold in the jurisdictions of New South Wales, Victoria and the Australian Capital Territory [36]. Informed by the model-based analyses presented here, state governments imposed strict stay-at-home measures (corresponding to high PHSMs) before relaxing those measures (to settings corresponding to approximately medium and then low PHSMs) upon reaching the 70% and 80% coverage thresholds, respectively. Assisted by the ongoing application of PHSMs and TTIQ, and greater than 80% vaccine coverage, the initial waves of Delta infection had peaked and were in decline by November 2021, with epidemic activity stabilizing at levels manageable within health system capacity—as anticipated by the scenarios explored here.

Response plans, and the modelling work supporting them, should be adaptable to new phases of the pandemic, including the emergence of new variants and intervention options. All findings from our scenario analysis were made in the context of the Delta variant (specifically, what was known in June 2021) and the Australian healthcare system and society under conditions of low prevalence. In the latter half of 2021, the modelling framework described here was adapted to investigate new epidemic dynamics and policy needs in response to emerging information on the increased severity of Delta (relative to ancestral strains), the emergence of Omicron, and the roll-out of third vaccine doses. The Omicron variant (BA.1) was first detected in Australia in late November 2021 and rapidly became the dominant circulating variant. At the time, daily case incidence in five of Australia's eight states/territories was either zero or fewer than tens of cases per day (electronic supplementary material, figures S21 and S22). Consequently, when widespread transmission became established at various time points beyond the 70% coverage threshold in late 2021/early 2022, the first ever SARS-CoV-2 epidemics managed by these jurisdictions were dominated by Omicron. Additional scenario analyses required adjustment to consider emerging evidence of Omicron's increased intrinsic transmissibility, higher propensity for immune-evasion, and decreased clinical severity relative to the Delta variant. These analyses also incorporated a data-driven model of the relationship between neutralizing antibody levels (either infection or vaccine-induced) and protection against a range of outcomes over time since infection/vaccine administration, which exhibits waning of protection. Furthermore, our vaccine allocation scenarios were restricted to two-dose vaccination of the 16+ adult population. With approval of vaccines for those 5–16 years of age and third doses for the adult population by late 2021, additional research was required to assess the anticipated additional benefits of these programmes. The scenario modelling described here informed a stepwise and agile approach to the relaxation of COVID-19 response measures in Australia in the vaccine era.

## Data Availability

All code and input datasets required to perform the analyses are available at https://github.com/aus-covid-modelling/NationalCabinetModelling and https://zenodo.org/record/8191103 [37], respectively, except for the raw Australian COVID-19 vaccination data. These data were provided by the Australian Government Department of Health under a data sharing agreement and are not publicly available. However, we provide aggregated datasets containing the daily proportion of people at a national level by dose number, vaccine product and age group for each scenario. The data are provided in electronic supplementary material [38].
